# Musculoskeletal Pain During Late Adolescence: The Generation R Study

**DOI:** 10.1002/ejp.70244

**Published:** 2026-03-03

**Authors:** Rosemarijn van Paassen, Max A. van Kessel, Sita M. A. Bierma‐Zeinstra, Marienke van Middelkoop

**Affiliations:** ^1^ Erasmus MC University Medical Center Rotterdam the Netherlands

## Abstract

**Background:**

Musculoskeletal (MSK) pain is one of the most prevalent health issues among adolescents. This study aimed to evaluate the prevalence and characteristics of MSK pain in 17‐year‐old adolescents and to examine its associations with demographics, previous reporting of musculoskeletal (MSK) pain, lifestyle factors, and behaviour.

**Methods:**

Data were derived from a longitudinal birth cohort study. MSK pain prevalence, location, and characteristics were assessed using a questionnaire. Demographics, BMI, previously reported MSK pain, lifestyle factors, and behaviour were derived from questionnaires and measurements at follow‐up moments during early and late adolescence. Data were analysed using descriptive statistics and logistic regression techniques.

**Results:**

2537 participants were included at follow‐up, with a mean age of 18.7 (0.8) years. A MSK pain prevalence of 15.5% (*n* = 393) was found. Of these, 62.3% experienced daily pain. Median MSK pain duration was 21.5 weeks (Interquartile range: 4.8–103.2), with a mean pain score of 5.3 (0–10 scale). The most frequently reported locations for MSK pain were the lower back (28.0%), the knee (26.2%), and the upper back (24.2%). Multivariable logistic regression showed that MSK pain at age 13 years (OR 2.13; 95% CI 1.48; 3.07) and behavioural problems (OR 1.77; 95% CI 1.20;2.62) were associated with MSK pain at age 18.7.

**Conclusion:**

MSK pain is common in adolescents, with a high prevalence and a chronic aspect. The most prevalent locations of MSK pain were the back and the knee, with a large share of participants experiencing chronic complaints. Previous MSK pain and behavioural problems at young adolescence are associated with MSK pain at age 18.7 in boys and in the total study population.

**Significance Statement:**

Current knowledge on musculoskeletal pain development and changes during adolescence is lacking in the literature. We report the prevalence and characteristics of musculoskeletal pain in late adolescence in a large population cohort study (N = 2537) and, due to the longitudinal basis of our study, compared these incidences to musculoskeletal pain incidence at early adolescence.

## 1 Introduction

Musculoskeletal (MSK) pain is one of the most prevalent health issues among adolescents, contributing to a rising rate of disability‐adjusted life‐years (DALYs) worldwide and is already the third leading cause of DALYs among adolescents (Guan et al. 2023). MSK pain in adolescents can disrupt normal daily activities and social participation and is associated with a high financial burden (Espirito Santo et al. 2024; Roth‐Isigkeit et al. 2005). The reported prevalence of MSK pain in adolescence varies widely, ranging from 25% up to 76%, with a higher prevalence among girls (Andreucci et al. 2021; Chambers et al. 2024; Keeratisiroj and Siritaratiwat 2018; Rathleff et al. 2013; Rhee et al. 2005). A prospective cohort study showed that 18% of young adolescents reported MSK pain, with approximately 90% of those cases classifying as chronic MSK pain and almost half of the participants experiencing daily pain (van Leeuwen et al. 2024). Moreover, MSK pain in adolescence is associated with a risk of persistent or chronic pain in adulthood (Briggs et al. 2016). Given the high prevalence and the rising burden of MSK pain in adolescents, it is important to gain a better understanding of MSK pain in adolescents.

Many factors have been reported to be associated with the presence of MSK pain in adolescents. For example, being overweight and having a tall stature may contribute to increased joint loading and pain in the knees or lower back (Huguet et al. 2016; Paulis et al. 2014). The relationship between physical activity and MSK pain is complex and somewhat contradictory (Heikkala et al. 2019; Huguet et al. 2016). While moderate physical activity may be protective (Guddal et al. 2017), some studies suggest that regular exercise may actually increase the risk of MSK pain (Huguet et al. 2016). Psychological factors such as internalising and externalising behavioural issues, sleep disturbances, and emotional symptoms like anxiety and sadness have been linked to the presence of MSK pain (Andreucci et al. 2021; Harrison et al. 2014; Huguet et al. 2016). Furthermore, MSK pain at a young age is associated with higher body mass index (BMI), behavioural problems, and physical activity (van Leeuwen et al. 2024). Altogether, there are multiple physical (such as BMI, physical activity, previous pain) and psychosocial (such as behavioural problems and anxiety) factors associated with the presence of MSK pain during childhood and puberty (van Leeuwen et al. 2024). Nevertheless, the aforementioned factors lack longitudinal evidence during puberty and late adolescence.

Therefore, this study aims to evaluate the prevalence and characteristics of MSK pain in late adolescents in a large population‐based birth cohort and to identify longitudinal relationships between physical and psychosocial factors and MSK pain in late adolescents.

## 2 Method

### 2.1 Study Population

This study was conducted as part of the Generation R Study, a population‐based prospective cohort study that aims to investigate growth, development, and health from foetal life until young adulthood. The study population consisted of women with an expected delivery date between April 2002 and January 2006 residing in Rotterdam, the Netherlands. At the start of the study, 9749 children and their pregnant mothers were included in the cohort. At regular intervals during infancy, childhood, and puberty, participants were invited to the research center to fill out questionnaires and undergo physical examinations. A detailed account of the Generation R study cohort can be found elsewhere (Kooijman et al. 2016).

The Medical Ethics Committee of the Erasmus Medical Center, Rotterdam, approved the study, and written informed consent was obtained from all participants at the age of 17. At follow‐up at age 13, informed consent was obtained from all parents and participants aged 12 and older, as required by Dutch law.

**FIGURE 1:**
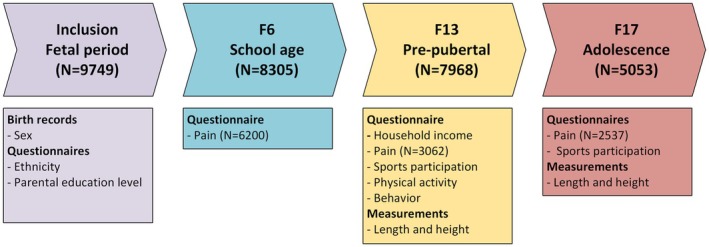
Timeline of the Generation R study with the data collected and used from each timepoint.

For the current study, questionnaire data from the inclusion period, as well as follow‐up at 6 (F6), 13 (F13), and 17 (F17) years, were included. Additionally, data from physical measurements taken at F13 and F17 were also included. See Figure 1 below for an overview of which data from which time point were included. The follow‐up moments aimed to collect data from participants at 13 and 17 years old; however, the actual age of the participants at that moment may differ, as measuring so many participants takes time. Additionally, the age of participants at F17 is much higher due to delays in measurements caused by the COVID‐19 pandemic.

### 2.2 Measurements—Questionnaires

#### 2.2.1 Demographics (Inclusion Period and F13)

Information on sex was obtained from midwives and hospital records at birth. Ethnicity and education level of parents were obtained from parental questionnaires at the time of inclusion, and household income was collected from questionnaires at F13. Ethnicity was based on the country of birth of both parents. The education level of the parents was obtained and categorised as high (higher education, master's degree), intermediate (higher education, bachelor's degree), and low (no higher education, primary school or secondary school). The net household income was derived, and the income was dichotomized into less than or more than €1600 per month based on the average net income in the Netherlands around 2014.

#### 2.2.2 Musculoskeletal Pain (F6, F13, and F17)

**FIGURE 2:**
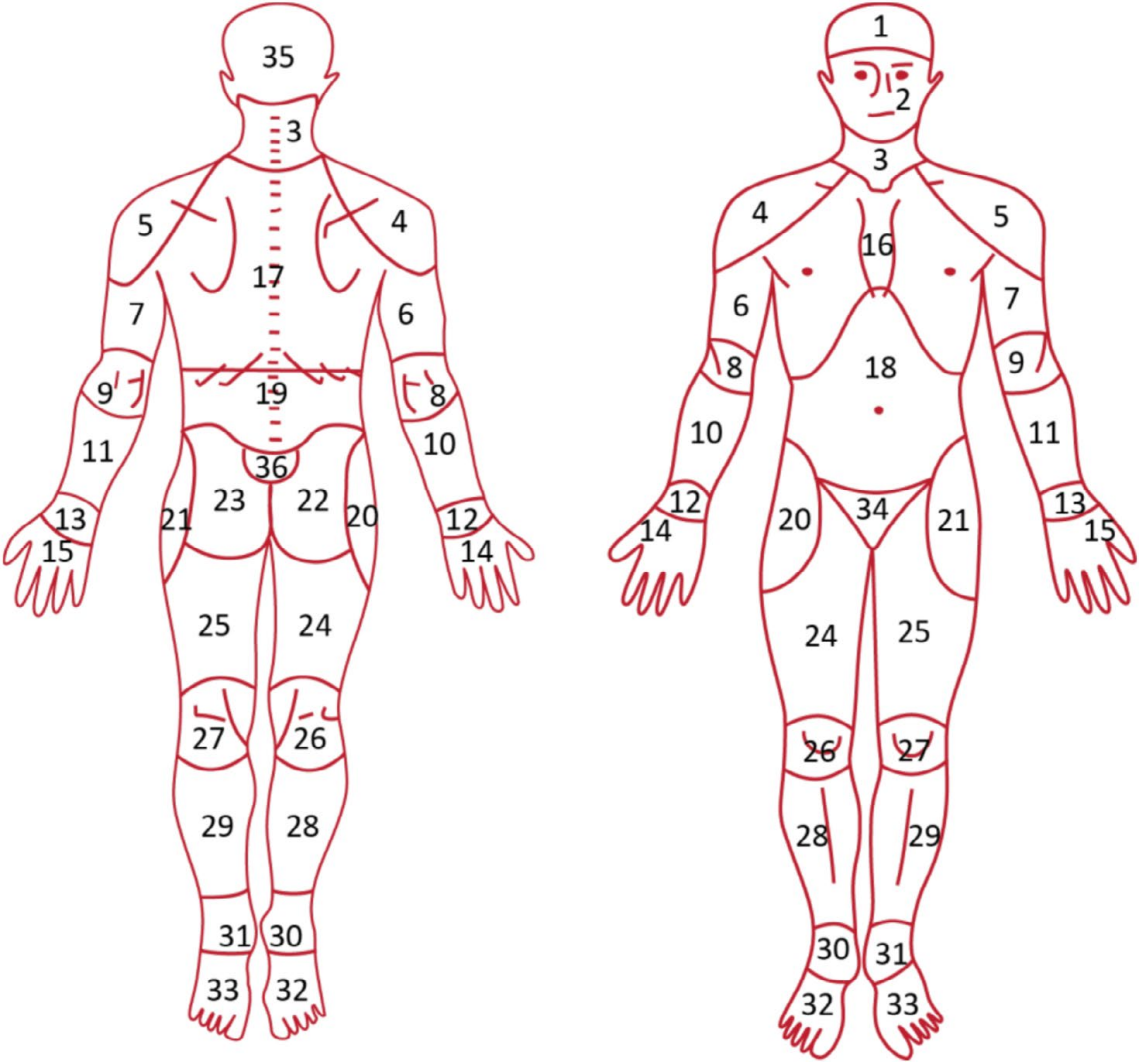
Pain mannequin with all 61 pain locations indicated on the front and the back of the body.

At 6 years old, the parents filled out questions regarding the pain of their child in the past 3 months in the following categories: head, stomach, back, arms or legs, neck, throat, ear, chest, and other. MSK pain at 6 years old was defined as pain in the neck, back, arms or legs. More extensive questionnaires on pain were completed at F13 and F17, which were identical in content and design. The participants were asked whether they experienced pain in the past 6 weeks. If so, the location of the pain was checked on a pain mannequin (Figure 2) with 61 possible locations, both at the front and back sides of the body. The locations of the neck, back, upper, and lower extremities were defined as MSK pain. All other pain locations were classified as “other pain”. Further questions about pain characteristics included the frequency of the pain (constant, (almost) daily, multiple times per week, weekly, monthly, or sometimes), pain duration (in weeks and/or months), the onset of the pain (suddenly or gradually), whether the pain was related to sports (yes or no), and pain intensity (numeric rating scale (NRS) from 0 to 10).

#### 2.2.3 Behaviour (F13)

Behaviour problems were assessed using the Child Behaviour Checklist (CBCL), a widely used, validated tool for assessing emotional and behavioural problems in children and adolescents (Achenbach and Edelbrock 1991). The total scores of internalising problems (Emotionally Reactive, Anxious/Depressed, Somatic Complaints, and Withdrawn scales), externalising problems (Attention Problems and Aggressive Behaviour scales), and a combined total score were calculated according to the instructions of the CBCL (Achenbach and Edelbrock 1991). To determine whether there is a (sub)clinical behavioural problem per subcategory, we compared the participants' scores with the cutoff scores of a Dutch reference group. The scores for internalising, externalising, and total problems were deemed (sub)clinical behavioural problems when they exceeded the 84th percentile (Tick et al. 2007).

#### 2.2.4 Activity Data (F13 and F17)

The in‐house designed questionnaires at F13 and F17 included questions on sports participation (dichotomized as yes or no), the type of transportation used to and from school, and the number of days they engage in physical activity for at least 1 h. Physical activity data were dichotomized into “not active” (being < 4 days of one‐hour activity per week) and “Physically active” (≥ 4 days with at least 1 h of activity per week). Active transportation was defined as at least one trip per week by foot or by bike.

### 2.3 Measurements—Physical Examination

#### 2.3.1 Height and Weight Measurements (F13 and F17)

Physical examination measurements were identical at F13 and F17 follow‐up. The participant's height was measured at the research center using a Harpenden stadiometer (Holtain Limited, Crymych, United Kingdom). The participant's weight was also measured at the research center using a mechanical weight scale (SECA, Hamburg, Germany) without wearing heavy clothing or shoes. Body mass index standard deviation scores (BMI‐SDS) adjusted for age and sex were calculated using the Dutch reference charts (Fredriks et al. 2000). Weight status was categorised following the cutoffs established by Cole and Lobstein (Cole and Lobstein 2012). Overweight was defined as being on the 90th percentile, corresponding to a BMI standard deviation score of approximately 1.3. Obesity was defined as being on the 98.9th percentile (Cole and Lobstein 2012). Additionally, change in BMI‐SDS between F13 and F17 was calculated (ΔBMI‐SDS).

### 2.4 Statistical Analysis

The characteristics of all participants at F17 were analysed using descriptive statistics for the total population and two subgroups (MSK pain, no MSK pain). The chi‐squared tests (for dichotomous data), analysis of variance (ANOVA) tests (for normally distributed continuous data), and Mann–Whitney *U*‐tests (not‐normally distributed continuous data) were performed to determine the statistical difference in characteristics between these two subgroups. Furthermore, the prevalence of MSK pain and frequencies of pain locations at F17 were determined. A chi‐squared test was used to analyse differences in the prevalence of the reported pain locations between boys and girls. The frequencies of MSK pain locations at F17 were compared with those at F13, using only participants with available data at both ages. In addition, multivariable logistic regression analysis was performed to test the association between characteristics associated with MSK pain at F6 or F13 and presence of MSK pain at F17. The multivariable logistic regression analysis was performed on a participant sample with complete data on all variables used in the analysis. Multicollinearity was tested using the variance inflation factor (VIF) and a correlation coefficient to ensure the accuracy of the multivariable logistic regression model. Variables that exhibited multicollinearity (correlation coefficient > 0.7 or a VIF > 10) were removed from the logistic regression model. The logistic regression analyses resulted in odds ratios (OR) with 95% confidence intervals (95% CI). All analyses were performed using SPSS software version 28.0.1.0. (Armonk, NY, IBM Corp). The level of statistical significance was defined as *p* < 0.05.

## 3 Results

### 3.1 Characteristics

The total study sample comprised 2537 participants at F17, with a mean age of 18.7 years, of whom 50.5% were girls, and 20.5% of participants were overweight or obese (Table 1). The ethnic demographic of the participants was predominantly Dutch, accounting for 64.2% of all participants, and a total of 8.5% of the participants were from low‐income households. Differences between children with and without MSK pain at F17 were found for sex, with girls more frequently reporting pain than boys (58.5% vs. 41.5%), and for parental education level, where lower levels of maternal (38.9% vs. 35.0%) and paternal education (43.4% vs. 35.1%) were more common in the MSK pain group. Children with MSK pain also more often came from lower‐income households, had a non‐Western ethnic background, showed higher weight status, and were more likely to report other types of pain compared to participants without MSK pain (Table 1).

**TABLE 1:** Participant characteristics of the total study population and participants with and without MSK pain.

	MSK pain (*n* = 393)	No MSK pain (*n* = 2144)	Total (*N* = 2537)	*p*
General data				
Sex				
Boys	163 (41.5%)	1092 (50.9%)	1225 (49.5%)	**< 0.001**
Girls	230 (58.5%)	1052 (49.1%)	1282 (50.5%)	
Maternal education				
Low	128 (38.9%)	671 (35.0%)	799 (35.6%)	**0.039**
Intermediate	109 (33.1%)	570 (29.8%)	679 (30.3%)	
High	92 (28.0%)	673 (35.2%)	765 (34.1%)	
Paternal education				
Low	129 (43.4%)	630 (35.1%)	759 (36.3%)	**0.003**
Intermediate	78 (26.3%)	445 (24.8%)	523 (25.0%)	
High	90 (30.3%)	720 (40.1%)	810 (38.7%)	
Ethnicity				
Dutch	218 (56.9%)	1310 (65.5%)	1528 (64.2%)	**0.008**
Other western	41 (10.7%)	179 (9.0%)	220 (9.2%)	
Non‐western	124 (32.4%)	510 (25.5%)	634 (26.6%)	
Low income, yes	36 (12.1%)	134 (7.9%)	170 (8.5%)	**0.016**
F6 data				
MSK pain at 6 years old, yes	36 (10.8%)	173 (9.2%)	209 (9.4%)	0.373
F13 data				
Age, years (SD)	14.0 (0.59)	13.9 (0.57)	13.9 (0.57)	0.175
BMI‐SDS	0.50 (1.22)	0.31 (1.12)	0.34 (1.14)	**0.004**
Overweight, yes	66 (18.3%)	251 (12.7%)	317 (13.6%)	**0.004**
Of which obesity, yes	14 (3.9%)	34 (1.7%)	48 (2.1%)	**0.008**
MSK pain, yes.	83 (31.2%)	214 (15.1%)	297 (17.7%)	**< 0.001**
Other pain, yes	29 (10.1%)	79 (5.6%)	10 (6.4%)	**0.001**
Sports participation, yes	235 (77.8%)	1506 (85.3%)	1741 (84.2%)	**0.001**
Physical activity > 4 days, yes	181 (69.1%)	1076 (68.8%)	1257 (68.9%)	0.938
Active transport to school, yes	224 (87.2%)	1378 (91.1%)	1602 (90.5%)	**0.048**
CBCL				
(Sub)clinical internalising problems, yes	74 (23.0%)	295 (16.0%)	369 (17.0%)	**0.002**
Internalising problem score	6.49 (6.14)	5.30 (5.42)	5.48 (5.55)	**< 0.001**
(Sub)clinical externalising problems, yes	58 (18.1%)	250 (13.6%)	308 (14.3%)	**0.032**
Externalising problem score	4.80 (5.71)	3.68 (4.60)	3.85 (4.80)	**< 0.001**
(Sub)clinical somatic problems, yes	52 (16.3%)	153 (8.3%)	205 (9.5%)	**< 0.001**
(Sub)clinical total problems, yes	71 (22.1%)	257 (14.0%)	328 (15.2%)	**< 0.001**
Total problems score	20.95 (17.80)	17.02 (15.02)	17.60 (15.54)	**< 0.001**
F17 data				
Age, years (SD)	18.7 (0.82)	18.7 (0.81)	18.7 (0.81)	0.733
BMI‐SDS	0.66 (1.29)	0.51 (1.23)	0.54 (1.24)	**0.031**
ΔBMI‐SDS (F17‐F13)	0.13 (0.84)	0.19 (0.80)	0.18 (0.80)	0.245
Overweight, yes	99 (25.2%)	418 (19.6%)	517 (20.5%)	**0.010**
Of which obese, yes	30 (7.7%)	113 (5.3%)	143 (5.7%)	0.063
Other pain	94 (45.2%)	114 (54.8%)	208 (8.2%)	**< 0.001**
Sports participation, yes	176 (44.8%)	1063 (49.6%)	1239 (48.8%)	0.569

*Note:* All values are given as mean (standard deviation) or *N* (%) unless otherwise specified. Bold values represent statistically significant differences (*p* < 0.05).

Abbreviations: MSK, musculoskeletal; NRS, numeric rating scale.

Children who reported MSK pain at F17 were more likely to be overweight or obese at F13, more often experienced pain at F13 (MSK or other types of pain), participated less often in sports at F13, and less often used an active form of transportation at F13. The MSK pain group also had higher scores on behavioral problems, including internalising, externalising, and total problem scores.

### 3.2 Pain Characteristics

**FIGURE 3:**
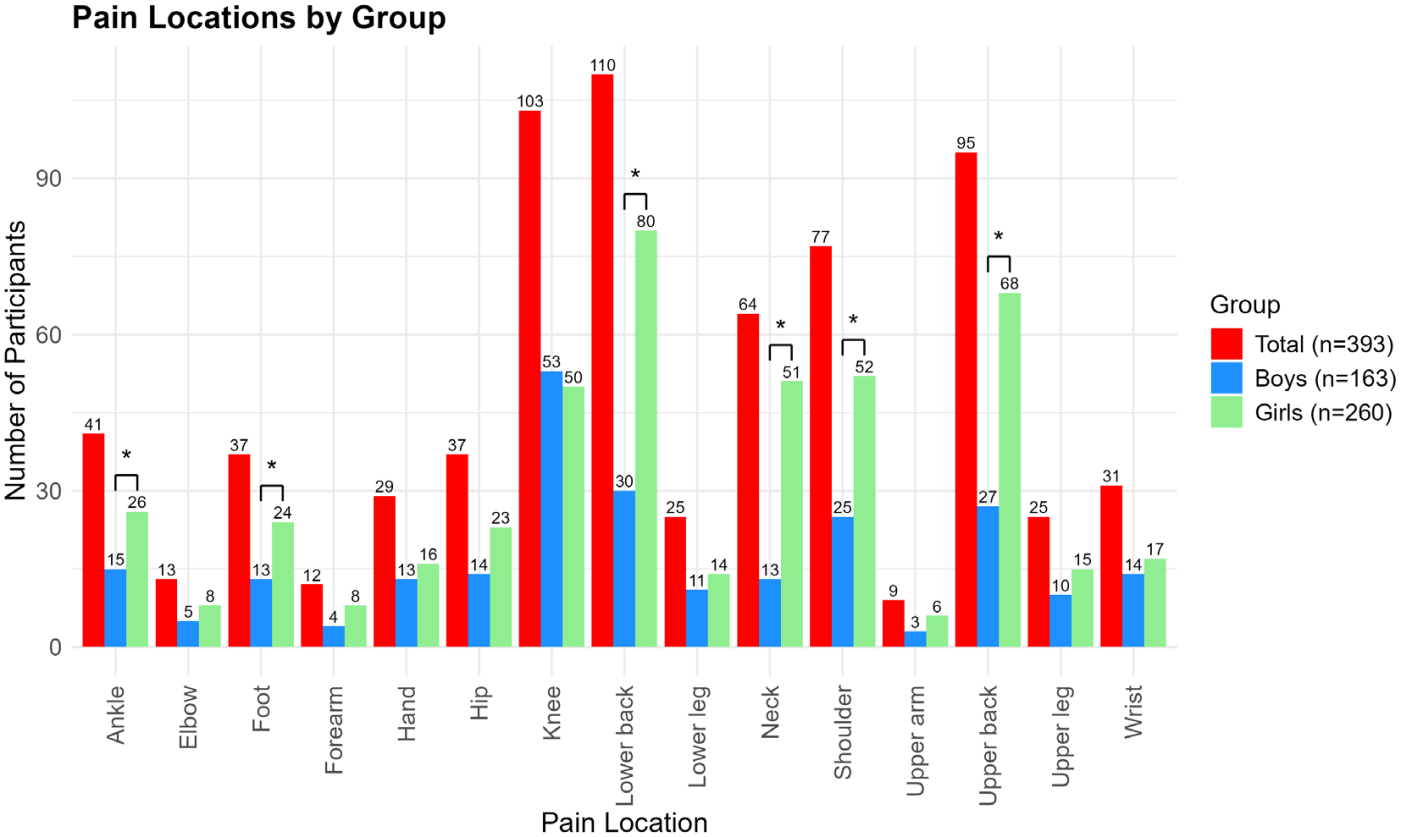
MSK pain locations of the 393 participants that reported MSK pain at age 17 for the total population, and for boys and girls separately. The total numbers are cumulative more than 393 because participants could report pain in multiple locations. *p < 0.05 indicating a significant difference between boys and girls.

**FIGURE 4:**
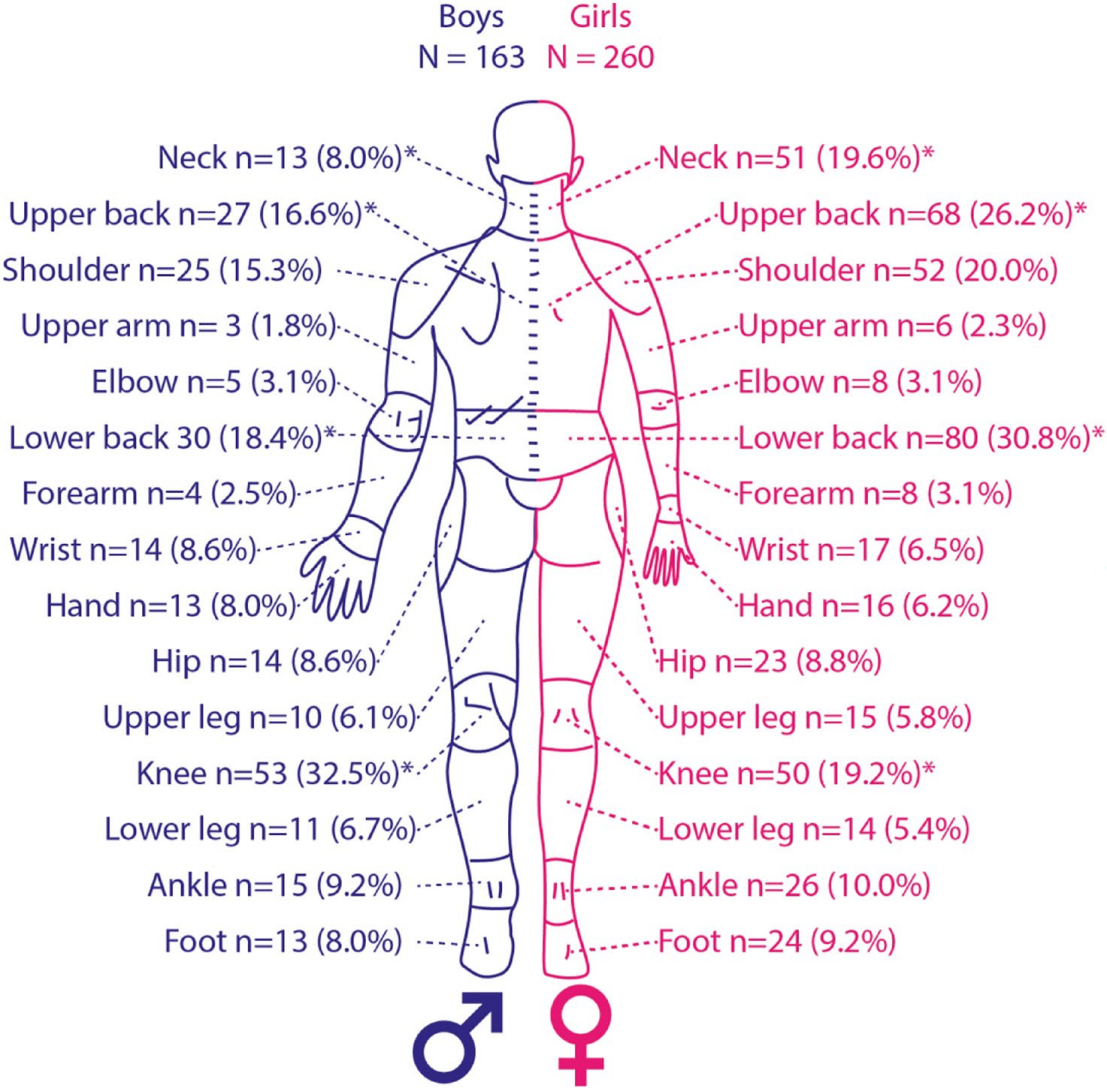
MSK pain locations of the 393 participants that reported MSK pain at age 17 shown for boys on the left and girls on the right. The number of participants with pain at each location and the percentage of boys/girls with pain in that location is shown. The percentage is calculated regarding the total number of boys or girls with pain. *p < 0.05 indicating a significant difference between boys and girls.

In total, 393 (15.5%) participants reported experiencing MSK pain in the past 6 weeks at F17. The most frequently reported locations of MSK pain were the lower back (28%), knee (26.2%), and upper back (24.2%). Significant differences in pain location between boys and girls were found for the upper back, lower back, knee, and neck (Figures 3 and 4).

Most participants with MSK pain reported pain at a single location (35.9%), followed by pain at two locations (33.1%), and pain at more than two locations (31.0%). 40.7% of participants with MSK pain experienced pain daily, and approximately half of the participants with MSK pain (47.8%) experienced pain related to sports. The median duration of MSK pain, chronicity of complaints, and pain intensity differed between boys and girls, with girls experiencing longer pain durations, more chronic complaints (lasting more than 3 months), and higher pain intensities (Table 2).

**TABLE 2:** Characteristics of MSK pain at age 17.

	Total population with MSK pain	Boys	Girls	*p*
	*n* = 393	*n* = 163	*n* = 230	
Number of MSK pain locations				
1 location	141 (35.9%)	76 (46.6%)	65 (28.3%)	< 0.001
2 locations	130 (33.1%)	48 (29.4%)	82 (35.7%)	
> 2 locations	122 (31.0%)	39 (23.9%)	83 (36.0%)	
Frequency^a^				
Constant	85 (21.5%)	31 (19.0%)	54 (23.5%)	0.836
Daily	160 (40.7%)	63 (38.7%)	97 (42.2%)	
Weekly	110 (28.0%)	47 (28.9%)	63 (27.4%)	
Monthly	8 (2.0%)	4 (2.5%)	4 (1.7%)	
Less than monthly	7 (1.8%)	4 (2.5%)	3 (1.3%)	
Pain onset^b^				
Suddenly	196 (51.2%)	95 (58.3%)	101 (43.9%)	0.002
Gradually	187 (48.8%)	61 (37.4%)	126 (54.8%)	
Pain related to sports, yes^c^	188 (47.8%)	96 (58.9%)	92 (40%)	< 0.001
Duration of complaints (weeks) (median, IQR)^d^	21.5[4.83–103.20]	10 [4.00–51.60]	34.40 [8.60–144.75]	< 0.001
Chronic pain (> 3 months), yes^e^	216 (56.8%)	70 (42.9%)	146 (63.5%)	< 0.001
Pain intensity score (NRS, 0–10)^f^	5.3 (1.7)	4.9 (1.8)	5.6 (1.6)	< 0.001

*Note:* Values are expressed as *n* (%), mean (SD) or median [Interquartile range].

^a^ Missing *N* = 23.

^b^ Missing *N* = 10.

^c^ Missing *N* = 11.

^d^ Missing *N* = 13.

^e^ Missing *N* = 13.

^f^ Missing *N* = 11.

**FIGURE 5:**
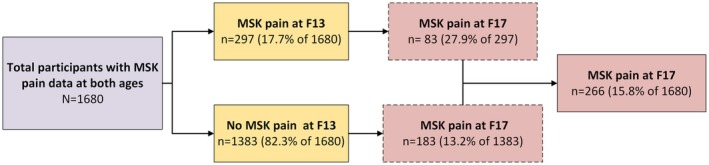
Flowchart of participants with MSK pain at F13 and F17 years old.

MSK pain data were available of 1680 participants at both F13 and F17 (Figure 5). Of these 1680 participants, 17.7% (*n* = 297) reported MSK pain at F13, and 15.8% (*n* = 266) reported MSK pain at age 17, of which 83 participants reported pain at both follow‐up moments. The prevalence of pain in the locations of the knee, lower back, and upper back differed the most between F13 and F17 (respectively: 46.8% vs. 28.9%; 10.4% vs. 25.6%; 6.1% vs. 30.1%). Only a few participants had pain in the same location at both ages (*n* = 24 of which 13 had knee pain at both moments). Among participants who reported MSK pain at F17, 31.2% (*n* = 83) experienced MSK pain at F13, of whom almost half (14.3%, *n* = 38) had previously reported knee pain.

### 3.3 Multivariable Analysis

Multivariable logistic regression analysis was performed on 1301 complete cases (Table 3), showing significant associations between MSK pain and behavioural problems at F13, and MSK pain at F17 in boys and the total study population. However, in girls, only MSK pain at F13 was significantly associated with MSK pain at F17.

**TABLE 3:** Multivariable logistic regression of MSK pain at focus 17 in the total study population, and boys and girls separately.

	Total (*N* = 1301)	Boys (*N* = 645) (12.2% MSK pain)	Girls (*N* = 656) (16.7% MSK pain)
General data			
Maternal education			
Low	Reference	Reference	Reference
Intermediate	1.28 [0.87; 1.88]	1.08 [0.60; 1.93]	1.43 [0.84; 2.42]
High	0.93 [0.62; 1.40]	0.68 [0.37; 1.25]	1.19 [0.69; 2.06]
Data at 6 years old			
MSK pain, yes	0.96 [0.57; 1.64]	0.59 [0.24; 1.46]	1.42 [0.71; 2.82]
Data at 13 years old			
BMI‐SDS	1.13 [0.98; 1.30]	1.08 [0.87; 1.34]	1.15 [0.95; 1.40]
MSK pain, yes	**2.13 [1.48; 3.07], *p* < 0.001**	**2.20 [1.23; 3.93], *p* = 0.008**	**2.00 [1.24; 3.21], *p* = 0.004**
Other pain, yes	1.36 [0.75; 2.47]	1.56 [0.53; 4.55]	1.29 [0.62; 2.71]
Sports participation, yes	0.73 [0.47; 1.14]	0.92 [0.44; 1.91]	0.65 [0.37; 1.14]
(Sub)clinical total behavioural problems, yes	**1.77 [1.20; 2.62], *p* = 0.004**	**2.03 [1.14;3.60], *p* < 0.001**	1.57 [0.92; 2.70]

*Note:* Logistic regression on complete cases. Results are presented as Odds Ratios (OR) with their 95% confidence interval (CI). Bold values represent statistically significant OR's (*p* < 0.05).

## 4 Discussion

In this study, the prevalence of MSK pain at 18.7 years old was 15.5%, with 56.8% having a chronic nature and with back and knee pain being the most reported pain locations. Girls experienced more MSK pain than boys. Additionally, our prognostic model emphasises the association between previous MSK pain and MSK pain at 18.7 years old and, interestingly, highlights a difference between the association in behavioural problems between boys and girls.

### 4.1 Comparison of Prevalence and Locations

A systematic review combined data from multiple studies and found a prevalence of chronic MSK pain of 25.7% (95% CI ranging from 17.3% to 35%) (Chambers et al. 2024). The prevalence of MSK pain at age 18.7 found in our study is lower than the prevalence reported in this review, which could be due to the participants age (0–19 years old, with a mean of 13.4, SD = 2.4 years) included in the review. Furthermore, the prevalence of MSK pain found at age 18.7 was also lower than the prevalence in the same population‐based birth cohort at F13, mean age 13.8 (15.5% vs. 23.3%) (van Leeuwen et al. 2024), which could be due to selection bias. The prevalence at F13 aligns with the prevalence reported in the systematic review by Chambers et al. (2024) (van Leeuwen et al. 2024). The lower prevalence at a later age may be due to puberty‐related physical changes, such as growth spurts and increased skeletal maturation, which typically cause lower limb pain (Lehman and Carl 2017). Another remarkable difference between the study at F13 and our current study is the most prevalent pain locations, with the knee being the most common pain location at age 13.8 and the (lower and upper) back being the most common at age 18.7. The prevalence of back pain increased drastically during adolescence, which could be attributed to the sedentary lifestyle of modern society, as literature shows a positive association between sedentary behaviour and lower back pain (da Costa et al. 2022).

Next to differences in age between the study populations, MSK pain locations and characteristics differed between boys and girls, with girls experiencing more overall pain, as well as specifically in the neck, shoulder, ankle, foot, upper back, and lower back, compared to boys. The knee remains a prevalent pain location (van Leeuwen et al. 2024), which could be explained by the chronic nature of non‐traumatic knee complaints in adolescence, such as patellofemoral pain syndrome (PFP) and Osgood‐Schlatter disease (Kastelein et al. 2015). These findings highlight the dynamic nature of MSK pain during adolescence and the importance of age‐ and sex‐specific approaches in research.

### 4.2 Factors Associated With Pain

Consistent with previous literature, we found an association between higher BMI or being overweight at F13 and the presence of MSK pain at F17 (Azabagic and Pranjic 2019; García‐Moreno et al. 2024; Paulis et al. 2014). The current understanding is that a higher BMI or being overweight results in higher joint loading and dysfunction, which can cause more joint injury or even joint misalignment (Molina‐Garcia et al. 2021), resulting in MSK pain. However, attributing MSK pain solely to mechanical loading is reductive, since adipose tissue is known to contribute to inflammation which may also play a significant role in the development and persistence of MSK pain (Walsh et al. 2018).

Our findings also suggest that lower levels of sports participation at age F13 were associated with increased MSK pain at F17. This finding aligns with the existing literature, which demonstrates a protective effect of sports participation against MSK pain (Guddal et al. 2017). However, the relationship between physical activity and MSK pain remains inconclusive, as neither our measurements of physical activity at F13 nor sports participation at F17 were significantly different between the MSK and no MSK pain groups. While high amounts of physical activity have been associated with higher rates of MSK pain in different studies (Briggs et al. 2016), others have shown no clear association (Auvinen et al. 2008; Maillane‐Vanegas et al. 2022; Rathleff et al. 2013). This inconsistency could be due to significant heterogeneity in how physical activity is defined and measured in different studies. Factors such as intensity, duration, and type (e.g., endurance versus loading) are often not specified, making comparison difficult. In our current study, we only collected data on the number of days participants engaged in physical activity for at least 1 h. We do not have information on the total number of hours spent on physical activity each week or the time spent on sports (categorised simply as participation: yes or no). Additionally, we did not assess the intensity of the activities or the number of days each week that participants took part in sports. More details on physical activity, such as the intensity, should be included in future studies to fully assess the association between different gradations of physical activity and MSK pain.

This study demonstrated an association between behavioural problems at young adolescence and MSK pain at 18.7 years old, which aligns with the established understanding that such problems and MSK pain are linked (Andreucci et al. 2021; Jussila et al. 2014; Larsson and Sund 2007; Vargas‐Prada and Coggon 2015). Especially, externalising problems such as attention deficit hyperactivity disorder (ADHD) have previously been identified to be associated with MSK pain (Andreucci et al. 2021). Interestingly, the multivariate logistic regression model showed a difference in the association between behavioural problems between boys and girls, with behaviour being associated with MSK pain in boys but not in girls. While we see a difference in the multivariate regression models, this does not necessarily mean that the effect of behaviour on pain only exists in boys. Boys more often show externalising problems (Table A2), which could increase risk taking behaviour, contributing to MSK pain. While girls more often show internalising problems (Table A2), such as depression or anxiety, which could also be related to MSK pain conditions (Andreucci et al. 2021). In addition to our primary analysis, we tried to enlarge our understanding by examining differences in CBCL subscores between boys and girls (Table A2). While some differences are observed, in both boys and girls higher percentages of behavioural problems across all subscores are observed in participants with MSK pain compared to participants without MSK pain. Although a clear association exists between MSK pain and behaviour, causality remains unclear. MSK pain may originate from or be influenced by psychological and behavioural issues, such as externalising and internalising problems (Andreucci et al. 2021). In contrast, there is also evidence that chronic pain is associated with structural and functional neuroplasticity in parts of the central nervous system as persistent pain alters connectivity, grey matter, and the functioning of neural circuits shared by pain and emotion processing (Jaffal 2025). In this pathway, chronic pain could potentially contribute to psychological symptoms, particularly internalising issues like depression and anxiety (Yang and Chang 2019). These contradictory findings emphasise the complexity of the relationship between pain and psychological health.

Besides behavioural problems, sociodemographic factors, such as parental education level, household income, and ethnicity, were included in our analyses. Our results show an association between sociodemographic factors and the presence of MSK pain. This aligns with literature showing a relationship between poverty and pain, but also between poverty and the presence of behavioural problems (Chen et al. 2024; Tran et al. 2020). The association between poverty and pain could be partly explained by a poorer lifestyle, poorer access to healthcare, or health literacy (Chen et al. 2024). Though children in poverty are also more often overweight or obese, which is related to an increased risk of pain (Walsh et al. 2018). This highlights the complexity of the relationship between sociodemographic factors and (MSK) pain, which goes beyond the scope of this manuscript.

### 4.3 Strengths and Limitations

The major strengths of this study are the large sample size and the wide range of variables available at different time points. The large sample size enhances the external validity of this study. However, some limitations need to be addressed.

There was a significant loss of participants from the questionnaires administered at F13 to those administered at F17. The group of participants who were lost at the follow‐up at F17 more often included participants with lower socio‐economic status and those who were overweight at age 13, compared to the total group of participants in this study (Table A1). This could have resulted in an underreported prevalence of MSK pain in this study and some bias in the multivariate regression model.

Another limitation concerns the collection of data about MSK pain via self‐reported questionnaires at a specific time point. Participants were required to answer whether they experienced pain in the last 6 weeks. Because of potential recall bias, this study could again underestimate the prevalence of MSK pain. The data about the frequency of the reported MSK pain show that most pain is experienced constantly or daily, and far less pain is experienced weekly or monthly, possibly due to recall bias.

## 5 Conclusion

MSK pain is common in adolescents, with a prevalence of 15.5% and more than half being chronic MSK pain, showing that MSK pain is a significant contributor to health problems in this population. The most prevalent locations of MSK pain were the back and the knee. Previously reported MSK pain and behavioural problems are predictive of having MSK pain at age 18.7 in the total population and boys, while in girls, only previously reported MSK pain was found predictive.

## Author Contributions

The study was designed by Marienke van Middelkoop and Sita M.A. Bierma‐Zeinstra. Max A. van Kessel performed data cleaning supervised by Rosemarijn van Paassen and Marienke van Middelkoop. Max A. van Kessel and Rosemarijn van Paassen performed data analysis and drafted the manuscript. Marienke van Middelkoop and Sita M.A. Bierma‐Zeinstra supervised data analysis. All authors provided feedback on the manuscript and approved the final version of the manuscript.

## Funding

The Dutch Arthritis Association (grant number: 21‐1‐204).

## Conflicts of Interest

The authors declare no conflicts of interest.

## Supporting information


**Data S1:** ejp70244‐sup‐0001‐DataS1.docx.
