# Supplementary material for: Musculoskeletal Pain During Late Adolescence: The Generation R Study
Source: Eur J Pain. 2026 Mar 3;30(3):e70244. doi: 10.1002/ejp.70244 (PMC12955511; doi:10.1002/ejp.70244)
Supplement: Supplementary file 1 — Data S1: ejp70244‐sup‐0001‐DataS1.docx. [file EJP-30-0-s001.docx]

Supplementary table

**Table A1: Differences in study population characteristics between the included participants at F17 and the participants lost to follow-up between F13 and F17**

|  | Total data present at F13 | Total data at F17 |
| --- | --- | --- |
| Sex  Boys   - Girls | 47.2%  52.8% | 49.5%  50.5% |
| Overweight, yes | 23.0% | 20.5% |
| Obesity, yes | 7.4% | 5.7% |
| Maternal education   - Low - Intermediate - High | 43.1%  28.0%  28.8% | 35.6%  30.3%  34.1% |
| Paternal education   - Low - Intermediate - High | 41.8%  22.3%  35.9% | 36.3%  25.0%  38.7% |
| Income, Low | 13.5% | 8.5% |
| Ethnicity   - Dutch - Other western - Non-western | 57.7%  8.1%  34.2% | 63.9%  9.2%  26.9% |
| *Data at F13* |  |  |
| MSK pain at F13, yes | 19.2% | 17.7% |
| Overweight, yes | 18.9% | 13.6% |
| Obesity, yes | 4.7% | 2.1% |
| Movement< 4 days, yes | 64.8% | 68.9% |
| Active transport school, yes | 86.3% | 90.5% |
| Sports participation, yes | 84.2% | 84.2% |
| (Sub)clinical internalizing problems, yes | 20.3% | 17.0% |
| (Sub)clinical externalizing problems, yes | 16.9% | 14.3% |
| (Sub)clinical somatic problems, yes | 13.3% | 9.5% |
| (Sub)clinical total problems, yes | 18.6% | 15.2% |

Table A2: Differences in characteristics between boys and girls in participants with and without MSK pain

| ***CBCL***  **Boys**  **Girls** | **MSK pain**  **(n=163)**  **(n=260)** | **No MSK pain**  **(n=1092)**  **(n=1022)** | **Total**  **(n=1255)**  **(n=1282)** | **P-value** |
| --- | --- | --- | --- | --- |
| (Sub)clinical internalizing problems, yes  Boys  Girls | 20 (16.0%)  48 (18.5%) | 127 (11.6%)  168 (16.4%) | 153 (12.2%)  216 (16.8%) | 0.052  **0.040** |
| Internalizing problems  Boys  Girls | 6.11 (6.08)  7.67 (6.19) | 4.90 (4.96)  5.72 (5.84) | 5.05 (5.12)  5.90 (5.91) | **0.011**  **0.027** |
| (Sub) clinical externalizing problems, yes  Boys  Girls | 25 (15.3%)  33 (12.7%) | 147 (13.5%)  103 (10.1%) | 172 (13.7%)  136 (10.6%) | 0.286  **0.026** |
| Externalizing problem score  Boys  Girls | 5.01 (5.71)  4.66 (5.72) | 3.97 (4.70)  3.38 (4.48) | 4.10 (4.84)  3.61 (4.74) | **0.022**  **<0.001** |
| (Sub)clinical somatic problems, yes  Boys  Girls | 13 (8.0%)  39 (15%) | 58 (5.2%)  95 (9.3%) | 71 (5.7%)  134 (10.5%) | 0.097  **<0.001** |
| (Sub)clinical total problems, yes  Boys  Girls | 27 (16.6%)  44 (16.9%) | 137 (12.5%)  120 (11.7%) | 164 (13.1%)  164 (12.8%) | 0.074  **<0.001** |
| Total problems score  Boys  Girls | 21.05 (16.89)  20.90 (18.45) | 17.72 (14.96)  16.3 (15.09) | 18.13 (15.24)  17.08 (15.81) | **0.019**  **<0.001** |

All values are given as mean (standard deviation) or N (%) unless otherwise specified. Abbreviations; MSK = Musculoskeletal
